# The 3D Tele Motion Tracking for the Orthodontic Facial Analysis

**DOI:** 10.1155/2016/4932136

**Published:** 2016-12-01

**Authors:** Stefano Mummolo, Alessandro Nota, Enrico Marchetti, Giuseppe Padricelli, Giuseppe Marzo

**Affiliations:** ^1^Department of Life, Health & Environmental Sciences, University of L'Aquila, L'Aquila, Italy; ^2^Department of Dentistry, PhD school, University of Tor Vergata, Rome, Italy

## Abstract

*Aim.* This study aimed to evaluate the reliability of 3D-TMT, previously used only for dynamic testing, in a static cephalometric evaluation.* Material and Method.* A group of 40 patients (20 males and 20 females; mean age 14.2 ± 1.2 years; 12–18 years old) was included in the study. The measurements obtained by the 3D-TMT cephalometric analysis with a conventional frontal cephalometric analysis were compared for each subject. Nine passive markers reflectors were positioned on the face skin for the detection of the profile of the patient. Through the acquisition of these points, corresponding plans for three-dimensional posterior-anterior cephalometric analysis were found.* Results.* The cephalometric results carried out with 3D-TMT and with traditional posterior-anterior cephalometric analysis showed the 3D-TMT system values are slightly higher than the values measured on radiographs but statistically significant; nevertheless their correlation is very high.* Conclusion.* The recorded values obtained using the 3D-TMT analysis were correlated to cephalometric analysis, with small but statistically significant differences. The Dahlberg errors resulted to be always lower than the mean difference between the 2D and 3D measurements. A clinician should use, during the clinical monitoring of a patient, always the same method, to avoid comparing different millimeter magnitudes.

## 1. Introduction

During the last years, three-dimensional (3D) imaging techniques have been developed, gaining a precious place in any field of dentistry [1, 2] and especially in orthodontics [3].

Over the years, orthodontic diagnosis and treatment planning were based essentially on 2-dimensional (2D) static imaging techniques that cannot give information about deepness of craniofacial structures [4, 5].

Inappropriate orthodontic treatment can produce adverse results and it is essential that full examination of skeletal form, soft tissue relationships, and occlusal features are performed prior to undertaking treatment. Lateral cephalograms are above all the predominantly used radiographic tool in orthodontics. They are the standard diagnostic tools in orthodontic diagnosis, the study of growing process, and control of treatment outcome. But posteroanterior X-rays are a more valid diagnostic tool for craniofacial asymmetries and transverse deficiencies. Trauma, cleft lip and palate, and unilateral condylar or mandibular hypertrophy are additional indications for posteroanterior view. The benefits gained from studying these radiographs range from assisting the orthodontist during diagnosis, as a tool to study growth in an individual through superimposition of structures on a longitudinal basis, and during evaluation of orthodontic treatment results.

In order to precisely replicate and describe the anatomy of the interested structures, 3D imaging has been applied in orthodontics to evaluate and record size and form of facial soft and hard tissues and dentition [6].

In 3D imaging evaluations stereophotogrammetry could be applied. Stereophotogrammetry consists in photographing a 3D object from 2 different coplanar planes in order to acquire a 3D reconstruction of the images. This technique has proven to be effective in the face display. Two cameras are configured as a stereo pair and are used to recover the 3D distance to features of the surface of the face [7]. Its software is therefore able to dynamically calculate geometric relationships between different facial points producing a 3D image and graphically represent their movements. [8]. The equipment is also able to calculate angles, distances, and associated kinetic variables [9].

Placing markers by using double-sided adhesive film, gel, or other ways in predetermined anatomical landmarks, it is possible to follow their movement in real time. The markers must be covered with reflective material. This instrument is now one of optoelectronic systems for medical use more technologically advanced and meets all the necessary requirements for cephalometric analysis: it is presented as noninvasive, does not create space because it uses passive markers, and does not interfere with the natural movement of the head of the subject or with the functionality of the soft tissues.

The vision system is composed of (i) two photoreceivers placed at the ends of the device, necessary for the three-dimensional reconstruction using stereophotogrammetric procedures; (ii) photoemitters that generate intermittently infrared light for the detection of the markers, and (iii) yellow LEDs that light up at the same time of photoemitters and allow seeing when the unit is in operation (Biomedical Sciences Research Institute 2008).

*Aim of the Study*. This pilot study aims to evaluate the applicability of 3D-TMT in a cephalometric evaluation of an orthodontic patient, comparing the data obtained with 3D-TMT with those obtained through a traditional cephalometric evaluation in frontal view.

## 2. Material and Methods

A group of 40 patients (20 males and 20 females; mean age 14.2 ± 1.2 years, range 12 to 18 years) in permanent dentition was included in the study. For each subject the measurements obtained by the 3D-TMT cephalometric analysis were compared with a cephalometric analysis carried out on radiographs in the frontal view.

### 2.1. Description of the Device

**Figure 1:**
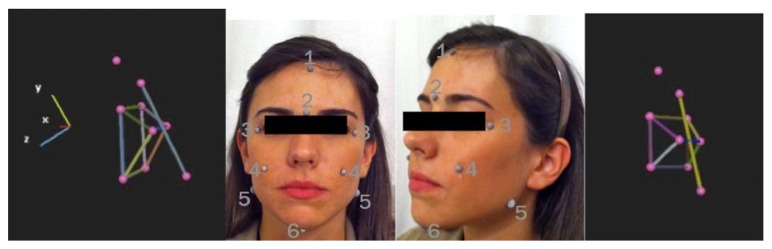
Reconstruction cephalometric digital with 3D-TMT and anatomical landmarks for placement of the markers: (1) Trichion; (2) glabella; (3) left and right frontozygomatic suture; (4) the most concave point of the left and right maxillary tuberosity (JL/JR); (5) left and right gonion; (6) menton.

The 3D Tele Motion Tracking (3D-TMT, MS Webcare, Division of Microsystems Srl, Milan, Italy) (Figure 1) is composed by a unit of vision, a telescopic support, and a tripod. The vision area can be chosen by varying the resolution used by the cameras: the sensors of the cameras can operate at different resolutions, that is, using various numbers of pixels. Higher resolutions allow fields of vision larger but, at the same time, lower frequencies of acquisition (fps); fps represents the maximum number of frames that the camera is able to capture every second. The typical frequency of acquisition (fps) of the images by the photoreceivers is 30 hz. The equipment is able to calculate angles, distances, and associated kinetic variables placing reflective markers by using double-sided adhesive film, gel, or other ways in predetermined anatomical landmarks; it is also possible to follow their movement in real time.

### 2.2. Preliminary Study on the Accuracy of the Instrument

For the assessment of the accuracy of the instrument, static tests were carried out preliminarily measuring known distances (15 cm) between markers placed on a rigid and static bar. The average values measured during the static tests and the related standard deviations were compared. The accuracy of the instrument denotes the closeness of computations or estimates to the exact or true values. A measurement is said to be more accurate when it offers a smaller measurement error. The accuracy was measured quantitatively by using relative error:

(1) $$\begin{array}{c} relative\;\;error=\frac{measured\;\;value-expected\;\;value}{expected\;\;value}. \end{array}$$

The accuracy has been established because the relative errors were lower than 1/100 of the expected values.

### 2.3. Outcomes

A 3D-TMT static evaluation was performed. Passive reflective markers with a diameter of 6 mm were used and the subject was placed at a distance of 2 meters from the photoreceiver for proper detection. The position of the markers was registered by the optoelectronic system.

**Figure 2:**
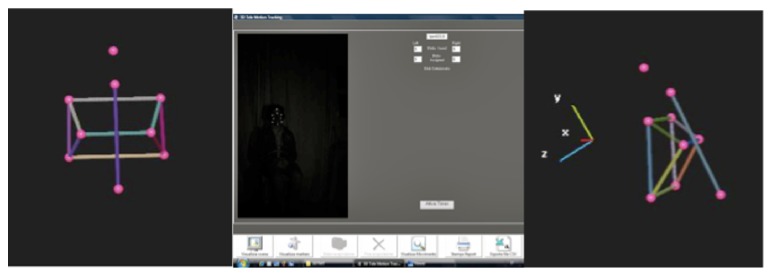
The three-dimensional reconstruction with 3D-TMT and scanned image in the 3D-TMT.

Eight markers were positioned on the skin of the face for the detection of the face (Figure 2). These were chosen as the most common points used for the frontal cephalometric analyses [10–12]:

The anatomical landmarks chosen for the detection were

trichion: point where the hairline meets the midpoint of the forehead,
glabella: point of smooth elevation of the frontal bone just above the bridge of the nose,
left and right frontozygomatic suture (ZL/ZR),
most concave point of the left and right maxillary tuberosity (JL/JR),
left and right gonion and menton.

The following planes were analyzed [10–12]:

- Median sagittal plane: derived from the intersection between glabella point and menton point; when there is facial symmetry, this plan allows to define the problems of midline deviation and lateral deviations
- Dentomaxillary frontal plan or zygomatic plane JL-AG or JR-GA: it is the reference plane of the molars in relation to their maxillary and mandibular bases
- Frontofacial plan ZL-AG or ZR-GA: it is the reference plane of the maxillary base that allows the differential diagnosis between dental or skeletal cross-bites
- Orbitofrontal horizontal plane ZL - ZR: plane between the right and left frontozygomatic sutures
- Horizontal plane JL - JR: plane passing between the concave points of the maxillary tuberosities
- Horizontal mandibular AG - GA or antegonial plan: passing between the right and left gonion.

The examination was performed with the patient sitting in front of the 3D-TMT.

### 2.4. Analysis of Data

Data are described as mean and SD.

Student's *t*-test for independent sample was performed to compare mean and SD of the variables measured with the traditional and the 3D-TMT methods, because the preliminary Kolmogorov-Smirnov *Z* attested a normal distribution of data (*Z* varied from 1.03 to 1.08; *p* varied from 0.15 to 0.19). For each test, *p* was set at 0.05 level.

A Pearson's correlation coefficient was also calculated between traditional and 3D-TMT measurements.

To detect any random error which may be of relevance, a proper analysis of the method error between the two recording methods was performed to quantify any random error (Dahlberg formula), in which the Dahlberg error, *D*, is defined as

(2) $$\begin{array}{c} D=\sqrt{\overset{N}{\underset{i=1}{\sum }}\frac{d_{i}^{2}}{2N}}, \end{array}$$

where *d*_*i*_ is the difference between the first and second measure and *N* is the sample size which was remeasured.

## 3. Results

The cephalometric results, carried out with 3D-TMT and with traditional posterior-anterior cephalometric analysis, are shown in Table 1. Mean values recorded using the 3D-TMT system are slightly higher than the values measured on radiographs. After application of Student's *t*-test. significant differences were observed between the measurements obtained with traditional methods and the 3D-TMT method.

Correlation analysis conducted using Pearson's correlation coefficients showed high correlations between traditional and 3D-TMT variables.

The Dahlberg formula gave results that are reported in Table 2.

For each variable, the relative form of Dahlberg error (RDE) was also reported in Table 2 as the proportion of Dahlberg error on the average difference between two comparative measures: RDE = Dahlberg error/mean of difference between two corresponding measurements.

For each variable, the Dahlberg errors resulted to be always lower than the mean difference between the 2D and 3D measurements, but in proportion, they resulted to be about 67.8%–71% of the average difference.

## 4. Discussion

The 3D-TMT can be used to conduct, through reflective markers placed at specific anatomical points, a cephalometric analysis on 3D plans for the study of an orthodontic patient, with the assessment of facial symmetry even at different depths within the craniofacial complex. The analysis with 3D-TMT also allows a dynamic and prognostic evaluation of the growth process of the patient. The evaluation with 3D-TMT is based on the localization of anatomical points on the skin surface of the face, analyzing the subject “aesthetics” and giving an important role to the soft tissues [13, 14]. During the last years many clinicians and authors underlined the underestimated importance of the soft tissues in the orthodontic treatment and a main aspect to be taken into consideration for judging the success of the treatment itself [15]. The analysis with 3D-TMT seems to allow a dynamic and prognostic evaluation of the facial structure growth process of the patient without exposure to ionizing radiation (X-rays) and thus absolutely being noninvasive and not harmful to biological level with no risk for the health of the patient [16].

In full accordance with the as Low as Reasonably Achievable (ALARA) principle this feature could bring this system to a wider diffusion replacing the traditional radiographic analysis [16, 17].

An advantage is represented by a survey carried out without exposure of the patient to ionizing radiation (X-rays), since the device acquires the images of the markers illuminated intermittently with infrared light, through two vision systems. It is therefore a noninvasive analysis and not harmful to biological level; it poses no risk to the health of the patient and can be repeated several times without risks. For this reason the test can be repeated several times on the same patient without damage, allowing a longitudinal evaluation during growth and during orthodontic treatment. The device captures three-dimensional images with a great accuracy in the localization of anatomical landmarks used for cephalometric analysis. A limitation of the instrument is that objects able to reflect the infrared beam could interfere with the actual uptake of the markers; thus, before starting the analysis, all objects in the visual field of reflectors which can be obstacles and generate artifacts should be removed.

From the results obtained, both precision and accuracy of the 3D-TMT are satisfactory and highly correlated with those obtained with cephalometric analysis in frontal view; thus the 3D-TMT seems a reliable instrument to easily analyze the facial pattern with cephalometry.

As expected, there was a statistically significant difference between measures obtained with 3D-TMT and cephalometric measures on frontal radiographs; in fact, 3D-TMT calculates the distances between cutaneous landmarks, while cephalometry indicates distances between skeletal points, not considering the thickness of soft tissues [18, 19].

Nevertheless, these mean differences are ever smaller than 1 mm and the comparison between 3D-TMT and frontal cephalometric measures showed a high correlation, thus suggesting they can guide the clinician toward the same diagnosis about facial morphology. The method error analysis, performed with the Dahlberg formula, revealed that a random method error could be about 67.8–71% of the mean difference between 3D and 2D values.

Our data are in accordance with the hypothesis that although we are examining the soft tissue outline, this also gives an indication of the underlying skeletal pattern. Obviously the soft tissue thickness may vary and mask the A-P skeletal pattern to some degree but the underlying skeletal pattern is therefore often reflected in the soft tissue pattern.

3D-TMT could be used in conjunction with other diagnostic tests performed routinely in orthodontic check-up, providing a complete picture of the situation of facial components in their entirety, even during motion. In addition. by examining them at the various stages of therapy, this instrument could allow checking in real time the physiological response to any phase of treatment, allowing assessing its suitability, and can be used effectively for cephalometric analyses and “aesthetics” analysis of the soft tissues. The 3D-TMT could also be useful for the research protocols on the study of the growing development of adolescents, to avoid X-rays exposure.

The disadvantage of using a 3D capture system is that the positioning of some cutaneous landmarks such as gonion and frontozygomatic suture is difficult and usually defined by the bone shape and found by touching the subject's body [20]. Because we were concerned about this problem empirically in the early stage of this study, we paid special attention to the positioning of these landmarks. The clinician must always keep in mind this disadvantage of 3D capture system, in each actual clinical case. In recent years. X-rays computed tomography technologies have been used in skeletal morphology research [19] as this technology provides positional data of the face surface and skeleton of a living person. We expect to obtain more accurate data of measurements related to bony landmarks in the near future also with this 3D capture system.

This study aims to introduce the Tele 3D Motion Tracking, stereophotogrammetric equipment, capable of replacing the traditional two-dimensional cephalometric analysis performed on cephalometric skull in posterior-anterior projection. An advantage is represented by a survey carried out without exposure of the patient to ionizing radiation (X-rays), since the 3D system-TMT acquires through two vision systems the images of the markers passive reflectors illuminated intermittently with infrared light. It is therefore an analysis that is completely noninvasive and not harmful to biological level that poses no risk to the health of the patient, unlike what occurs in normal cephalometric radiography that is required. For this reason the test can be repeated several times on the same patient without damage, also allowing a longitudinal evaluation during growth and during orthodontic treatment, as, for example, after the palatal expansion, or during an orthopedic-functional treatment. Moreover, the 3D-TMT allows you to make the cephalometric analysis extremely quickly.

## 5. Conclusions

This study aims to introduce the Tele 3D Motion Tracking, a stereophotogrammetric equipment able to do a cephalometric study of the soft tissues. The recorded values obtained using the 3D-TMT analysis were correlated to cephalometric analysis, although the millimetric values have small but statistically significant differences, and the method error analysis revealed the possibility of high error values.

A clinician should use, during the clinical monitoring of a patient, always the same method (traditional cephalometric analysis or 3D-TMT analysis), to avoid comparing different millimeter magnitudes. With this recommendation, the 3D-TMT analysis seems a viable method for the study of facial morphology and its monitoring during the growth of a patient, or during a therapy.

Further studies should be carried out to evaluate results during motion and other orthodontic applications of the 3D-TMT in diagnosis and follow-up of an orthodontic treatment.

## Figures and Tables

**Table 1:** Difference between the values of the frontal cephalometric traditional analysis and 3D-TMT.

	2D		3D		Difference		*p* value	Pearson's correlation coefficient
	Mean	SD	Mean	SD	Mean	SD		
AG-PSM	53.68	9.63	54.44	9.76	−0.76	0.82	0.0000	0.997
GA-PSM	53.59	9.42	54.26	9.76	−0.68	0.91	0.0001	0.996
JL-AG	50.50	8.69	51.09	8.75	−0.59	0.96	0.0011	0.994
JL-PSM	36.09	3.32	36.71	3.45	−0.62	0.89	0.0003	0.966
JLZL	59.56	5.24	60.15	5.26	−0.59	0.78	0.0001	0.989
JR-GA	50.82	9.13	51.47	9.22	−0.65	1.01	0.0007	0.994
JR-PSM	35.74	3.70	36.35	3.69	−0.62	0.89	0.0003	0.971
JR-ZR	59.71	5.32	60.56	5.28	−0.79	0.55	0.0000	0.994
PSM	131.29	7.68	131.71	7.58	−0.41	0.99	0.0207	0.992
ZL-AG	91.59	14.08	92.21	14.15	−0.62	0.82	0.0001	0.998
ZL-PSM	49.50	4.24	50.26	4.40	−0.76	0.92	0.0000	0.978
ZR-GA	96.18	14.65	96.71	14.69	−0.53	0.79	0.0251	0.999
ZR-PSM	47.26	5.24	47.85	5.34	−0.59	0.89	0.0005	0.986

PSM = Sagittal median plane.

JL-AG = Distance between JL and AG points.

JR-GA = Distance between JR and GA points.

ZL-AG = Distance between ZL and AG points.

ZR-GA = Distance between ZR and GA points.

ZL-PSM = Distance between ZL point and PSM.

ZR-PSM = Distance between ZR point and PSM.

JL-PSM = Distance between JL point and PSM.

JR-PSM = Distance between JR point and PSM.

GA-PSM = Distance between GA point and PSM.

AG-PSM = Distance between AG point and PSM.

JL-ZL = Distance between JL and ZL points.

JR-ZR = Distance between JR and ZR points.

**Table 2:** Method error analysis.

	2D		3D		Difference		$\;\overset{}{\underset{}{\sum }}d^{2}$ (*n* = 40)	$D=\sqrt{\overset{N}{\underset{i=1}{\sum }}\frac{d_{i}^{2}}{2N}}$	RDE (relative form of Dahlberg error)
	Mean	SD	Mean	SD	Mean	SD			
AG-PSM	53.68	9.63	54.44	9.76	−0.76	0.82	22.63	0.53	70%
GA-PSM	53.59	9.42	54.26	9.76	−0.68	0.91	18.94	0.48	70.6%
JL-AG	50.50	8.69	51.09	8.75	−0.59	0.96	13.26	0.4	67.8%
JL-PSM	36.09	3.32	36.71	3.45	−0.62	0.89	15.65	0.44	70.9%
JLZL	59.56	5.24	60.15	5.26	−0.59	0.78	13.21	0.4	67.8%
JR-GA	50.82	9.13	51.47	9.22	−0.65	1.01	16.2	0.45	69.2%
JR-PSM	35.74	3.70	36.35	3.69	−0.62	0.89	15.24	0.43	69.3%
JR-ZR	59.71	5.32	60.56	5.28	−0.79	0.55	24.26	0.55	69.6%
PSM	131.29	7.68	131.71	7.58	−0.41	0.99	6.83	0.29	70.7%
ZL-AG	91.59	14.08	92.21	14.15	−0.62	0.82	15.23	0.43	69.3%
ZL-PSM	49.50	4.24	50.26	4.40	−0.76	0.92	23.52	0.54	71%
ZR-GA	96.18	14.65	96.71	14.69	−0.53	0.79	11.21	0.37	69.8%
ZR-PSM	47.26	5.24	47.85	5.34	−0.59	0.89	13.78	0.41	70%
